# Correction: Protein fractional synthesis rates within tissues of high- and low-active mice

**DOI:** 10.1371/journal.pone.0248081

**Published:** 2021-02-25

**Authors:** Kristina M. Cross, Jorge Z. Granados, Gabriella A. M. Ten Have, John J. Thaden, Marielle P. K. J. Engelen, J. Timothy Lightfoot, Nicolaas E. P. Deutz

The image for Fig 1 is incorrect. Please see the correct version here.

**Fig 1:**
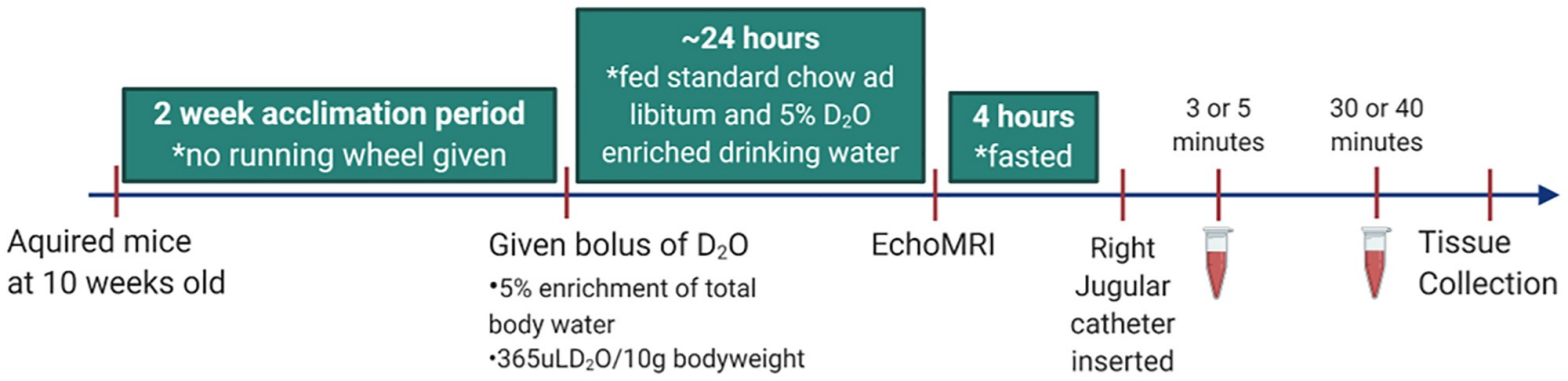
Timeline of study.
