# Supplementary material for: Protein fractional synthesis rates within tissues of high- and low-active mice
Source: PLoS One. 2020 Nov 30;15(11):e0242926. doi: 10.1371/journal.pone.0242926 (PMC7703944; doi:10.1371/journal.pone.0242926)
Supplement: Attachment — Submitted filename: Response to Reviewers.docx [file pone.0242926.s001.docx]

Ms. Ref. No.:  PONE-D-20-23160
Title: Protein fractional synthesis rates within tissues of high- and low-active mice

Dear editor and reviewers,

We thank the reviewers for their important and valuable time and comments. We have addressed these comments in the revised manuscript with track changes, made the required updates and therefore feel that it has improved the manuscript considerably.

Below are the point-by-point responses to each comment:

**Reviewer #1:**

**In this manuscript Cross et al. report the fractional synthesis rate of different tissues in two inbred mouse strains with different levels of physical activity. The manuscript is poorly written and does not follow the journal’s formatting guidelines.**

Response: Thank you for pointing this out and for your time spent reviewing our manuscript. The formatting has now been edited to follow journal guidelines and the flow has been improved.

**I find the premise that FSR drives physical activity puzzling. It would be easier (and testable) to argue that physical activity impacts FSR.**

Response: Our group has a long history of using physical activity as our dependent variable to investigate the biological factors that drive/influence physical activity. Thus, our study was an observational study aimed to view if there is a relationship between physical activity level and protein FSR of several tissues in high-active mice compared to low-active mice. We appreciate the hypothesis that you put forward, but others [1] have shown that using exercise as the training stimulus causes wide-spread biological alteration. Thus, since we hypothesized that there may be an inherent metabolic component that drives physical activity, we investigated whether protein FSR would be a potential driver.

**In addition to physical activity, the two inbred strains differ in other traits which may confound the interpretation of the results.**

Response: Yes we agree that the two inbred strains may differ in other traits. However, these two strains have been extensively used in similar studies considering genetic factors that alter physical activity over the past fifteen years, with their genomic, transcript, miRNA, and proteomic differences thoroughly cataloged. Recently, we have also explored plasma arginine production differences [2], which led us to examine protein FSR. It is possible that protein FSR would not drive physical activity, but this study’s results gives us a foundation to further hypothesize how protein FSR would alter physical activity. This is explained in the limitations section (lines 334-339).

**It was unclear why FSR was determined after surgery until it was mentioned in limitations that these results were part of another study.**

Response: We are sorry to have created this confusion. Blood was collected during surgery because this study was part of a larger metabolic analysis study. The explanation of surgery has been added to the methods section (lines 106-110).

**The feed removal, the anesthesia and the surgical procedure may have affected FSR without offering any advantage to its determination.**

Response: This is a correct observation. Although the food removal may have an effect on protein FSR, both groups had food removal so the effect should be the same. The anesthesia and surgical procedure would not make a large impact on the protein FSR as the incorporation of tracer from D_2_O has taken place over 24 hours. Therefore an increase or decrease in protein FSR due to the surgery would have only affected the last hour of a 24 hour measurement. A 20% reduction in the FSR during the last hour would only have about 1% effect on the measured FSR. In addition, all mice underwent the same type of surgery and we can anticipate that the effect on FSR would be roughly the same. This explanation has been added to the limitations section (lines 322-328).

**In addition the administration of fluids during surgery likely diluted the D2O enrichment.**

Response: We do acknowledge that an addition of fluids would dilute the D_2_O enrichment as the fluid that is adminsited has only natural occurring enrichment of D_2_O. This event then would reduce the alanine precursor enrichment in the last hour of a 24 hour presence of D_2_O. For example, if the unlabeled alanine TTRs is diluted in the last hour of a 24 hour measurement, that would reduce the FSR only a small fraction. If for instance, the enrichment is diluted by 12.5% (infused H_2_O 2 ml, total body water of the mouse 16 ml (about 65% BW mouse is H_2_O), the overall reduction in the alanine enrichment over 24h is 0.5%. In addition, we observe that enrichment of the blood taken at minute 3 before any saline was given (n=15; 0.1967 [0.1875, 0.2067]) vs the blood taken at minute 30 after the dilution (n=15; 0.1981 [0.1895, 0.2067]) was not statistically significantly different (p=0.487). Thus, we do not believe that the surgery makes an impact on the final results.

**Furthermore, the D2O enrichment reported in table 2 (0.2416) is outside the 95% CI of the values reported in the text. In fact, this value seems rather high if the authors successfully achieved a 5% D2O enrichment.**

Response: Thank you for catching this. The error has been corrected and all other data has been checked. The correct value for plasmaALA TTR is 0.2013 [0.1968, 0.2058].

**Some tables and figures repeat the same data (e.g., Table 4 and Fig. 2) and some other are redundant and unnecessary (e.g., Table 3 and Fig. 3).**

Response: Table 4 has been removed and figure 3 has been updated to include the q-values that were reported in the table. With the deletion of table 4, figure 2 can be kept for readers to visualize the relationship of protein FSR between organs and was moved to earlier in the manuscript in order to keep the flow from general to more specific data. Table 3 is necessary as we use these values to explain why we used plasma levels instead of intracellular values.

**Reviewer #2:**

**Summary. Experts in the application of tracer methods in studies of protein kinetics have examined whether physical activity is associated with fundamental differences in protein synthesis. Mice were studied using D2O and protein synthesis was measured in multiple tissues over a 24-hour period. Overall, there were no major differences except for kidney and a suggestion in lung. Although the data are largely negative (i.e. virtually no differences between groups), the study is generally well done but it seems like several choices in the protocol design should be better explained.**

Response: Thank you for your kind words and time spent reviewing our manuscript.

**Major comments.**

**If words permit, you might add a sentence or 2 in the ABSTRACT regarding a high-level view of the data. For example, values of protein synthesis in different tissues follow an expected pattern.**

Response: More data and additional conclusion information has been added to the abstract.

**The need for a surgery seems odd. It looks like you only collected blood? Is this necessary?**

Response: Sorry we did not make this clear. Blood was collected during surgery because this study was part of a larger metabolic analysis study. The explanation of surgery has been added to the methods section (lines 106-110).

**Also, there’s a peculiar statement in the 1st sentence under LIMITATIONS – you note that “…a pulse of stable isotope tracers …” was given ~ 1 hour before samples were collected. It looks like you had more going on in this study than simply giving D2O. It would be helpful to clarify the true protocol. What pulse of tracers?**

Response: This study was part of a greater study that administered a stable isotope tracer pulse in order to measure plasma amino acid production, clearance, and concentrations in plasma as reported in Granados et al. [2]. D_2_O was given to measure protein FSR and organ tissue was collected to measure amino acid concentration. The whole protocol of this metabolic kinetic study is aimed to answer the question of what determines physical activity level and specifically does a metabolic component determine physical activity level. The pulse of tracers is described in Granados et al. [2] and is not described in this paper because it was not used to create any of these measurements. A mention of the study being part of a greater study has been added to the methods (lines 106-110).

**Table 1 shows values for “total-body water” – the values are < 2 ml. What does this represent – intake? Please clarify.**

Response: Thank you for pointing this out. This value was an error which has been replaced with an estimate of total body water calculated as 74.7% of total body weight for each mouse [3]. Added in table 1 and line 104.

**It’s unfortunate that you did not directly measure body water labeling.**

Response: Thank you for your comments but we have learned from other studies that the alanine enrichments from water labelling are an inconsistent measurement as the enrichment ratio between alanine and D_2_O can range from 3.7 [4] [5] to 4.0 [6]. For this reason we chose to analyze the true precursor of the incorporation into protein alanine and not the indirect precursor D_2_O. By calculating the tracer-to-tracee ratio (TTR), we are able to report a more accurate enrichment in the precursor pool. We have added this information to the manuscript (lines 179-183).

**I can appreciate your attention towards the labeling of alanine in plasma vs respective tissues, indeed, alanine is the true precursor. That said, the plasma alanine labeling seems too high given the dose of tracer you’ve administered. For example, we might expect ~ 5% water enrichment which limits the alanine to ~ 18.5 or 20% (3.7 or 4 times), however, the plasma is ~ 24%.**

Response: Thank you for this observation. The originally reported value was incorrect. The error has been corrected in table 2. The correct value for plasmaALA TTR is 0.2013 [0.1968,0.2058].

**This seems too high, in fact, tissue enrichments are very much in line with the expectation of ~ 3.7-4x equilibration between water and alanine labeling. You might want to elaborate on this point.**

Response: The corrected and properly calculated value represents a ~20% enrichment value as you pointed out. All of the reported tissue protein FSR using the plasma alanine TTR were already calculated with the correct values.

**It’s also odd that the suppl data show plasma alanine ~ 20% in each group – how does each group reach ~ 20% in suppl data but the combined data in Table 2 seem to reach ~ 24%?**

Response: The supplemental data is correct while the combined data has now been corrected.

**The legend for Table 2 and 3 should more clearly state that you combined data for all animals.**

Response: Thank you for pointing this out and explanation of the combination has been added to the legends of each table.

**It might be better to change the order of Figure 2 and 3. In Figure 3 you get at more general trends in data / differences across tissues (comparable to what you show in Table 2 and 3) and then break data into active vs inactive groups (e.g. Table 4 comes last, why not make the data in Figure 2 become Figure 3)?**

Response: We have changed the order to better the flow. Thank you.

**Minor comments.**

**You might explain whether you think studying the animals during activity would have been better? For example, animals were effectively sedentary for ~ 2 weeks prior to the study.**

Response: As we noted in the response to Reviewer 1, we are concerned that exposure to physical activity would alter protein FSR [7] and gene expression [8]. We have previous data [9] that shows that exposure of the animals to a running wheel for just one week alters gene expression significantly. To prevent this potential alteration of gene expression - and potential alteration of FSR and protein expression - we have chosen to use naive animals from these well-characterized inbred strains (e.g. [10]). This design allows us to prevent the potential confounding of the protein FSR/protein expression responses by physical activity exposure. We added this explanation to the methods section (lines 80-86).

**Why not study subgroups who are regularly running vs those who are not? Can you comment on food intake when animals are exposed to running? It looks like there would be more marked effects if one group normally runs ~ 0.6 km/day vs another runs ~ 9.5 km/day. Not asking to do this experiment, just bring readers along with the rationale.**

Response: Thanks for this comment. We actually have done this study previously [11] and shown that there were differing food intake responses between the low active and high-active animals (due to supply issues, the low-active and high-active animals in the paper were of different strains, with similar activity levels as those in this current study). Because the low active animals did not exercise much, their food intake levels were similar to animals that did not have wheels. On the other hand, the high active animals, when given a running wheel, increased their food consumption with the increased caloric intake almost exactly matching the metabolic cost of the wheel running. As such, while the food intake may be slightly different between our high and low active strains, we believe this is accommodated in our consideration of the body weight of these animals. The reference to the study explaining food intake in physical activity levels has been added to the methods (lines 79).

**As well, you chose to study all animals in a fed state. Would it have been advantageous to randomize each group to overnight fed vs fasted protocols and look for phenotypes that way? Again, not asking for more studies, just outline your choices. You are addressing a very important problem (i.e. the interplay between lifestyle and metabolic regulation), the question/comment here is … would it be of value to probe this with a different experimental paradigm?**

Response: For the purpose of this study we observed the mice at a fasted state as a fed state will likely alter protein FSR values. In a fasted state, the mouse is in a steady state, because in a fed state, we expect the food to increase the tracee pool thus diluting the alanine TTR. In addition, the equations used to calculate protein FSR in the fastest state requires less assumptions than if in a fed state.

**You might want to explain why different collision energies are used for M0 (-68 V) vs M1 (-29 V)alanine.**

Collision energy (CE) was optimized in the alanine M1 MS/MS channel, but deoptimized for alanine M0 itself. This allowed more samples to be injected for better precision while measuring low-level M1 species while avoiding saturation of the ion-counting detector by the much more abundant alanine species. Because the same "detuning" was applied for external-standard calibration samples, the modeled calibration line removes the detuning factor. This explanation was added to the methods section (lines 151-155).

**It looks like low resolution figures are used here, the graphics are “fuzzy”. Presumably you will need to modify in finalized versions.**

Response: New graphics with higher resolution will be uploaded.

**It does not seem like you need suppl data, I would suggest you consider adding those data to the main paper.**

Response: The plasma alanine TTR and protein-bound alanine TTR data of each physical activity level has been converted from the supplemental table to a figure and added to the manuscript as figure 2A and 2B (lines 243-245).

References

1.Massett MP, Berk BC. Strain-dependent differences in responses to exercise training in inbred and hybrid mice. American Journal of Physiology-Regulatory, Integrative and Comparative Physiology. 2005;288(4):R1006-R13.

2.Granados JZ, Ten Have GAM, Letsinger AC, Thaden JJ, Engelen MPKJ, Lightfoot JT, et al. Activated whole-body arginine pathway in high-active mice. PLOS ONE. 2020;15(6):e0235095.

3.Annegers J. Total Body Water in Rats and in Mice. Proceedings of the Society for Experimental Biology and Medicine. 1954;87(2):454-6.

4.Wilkinson DJ, Franchi MV, Brook MS, Narici MV, Williams JP, Mitchell WK, et al. A validation of the application of D2O stable isotope tracer techniques for monitoring day-to-day changes in muscle protein subfraction synthesis in humans. Am J Physiol Endocrinol Metab. 2014;306(5):E571-E9.

5.Previs SF, Fatica R, Chandramouli V, Alexander JC, Brunengraber H, Landau BR. Quantifying rates of protein synthesis in humans by use of 2H2O: application to patients with end-stage renal disease. American Journal of Physiology-Endocrinology and Metabolism. 2004;286(4):E665-E72.

6.Brook MS, Wilkinson DJ, Smith K, Atherton PJ. The metabolic and temporal basis of muscle hypertrophy in response to resistance exercise. European Journal of Sport Science. 2016;16(6):633-44.

7.Phillips SM, Tipton KD, Aarsland A, Wolf SE, Wolfe RR. Mixed muscle protein synthesis and breakdown after resistance exercise in humans. 1997;273(1):E99-E107.

8.Dawes M, Kochan KJ, Riggs PK, Timothy Lightfoot J. Differential miRNA expression in inherently high- and low-active inbred mice. Physiological Reports. 2015;3(7):e12469.

9.Dawes M, Moore-Harrison T, Hamilton AT, Ceaser T, Kochan KJ, Riggs PK, et al. Differential gene expression in high- and low-active inbred mice. Biomed Res Int. 2014;2014:361048.

10.Lightfoot JT, Leamy L, Pomp D, Turner MJ, Fodor AA, Knab A, et al. Strain screen and haplotype association mapping of wheel running in inbred mouse strains. J Appl Physiol (1985). 2010;109(3):623-34.

11.Jung AP, Curtis TS, Turner MJ, Lightfoot JT. Physical activity and food consumption in high- and low-active inbred mouse strains. Med Sci Sports Exerc. 2010;42(10):1826-33.
